# Immune Thrombotic Thrombocytopenic Purpura in a Patient with Suspected COVID-19: Hydroxychloroquine Culprit or Just Happenstance?

**DOI:** 10.4274/tjh.galenos.2021.2021.0057

**Published:** 2021-06-01

**Authors:** Tajamul H. Mir

**Affiliations:** 1 Khyber Medical Institute Srinagar, Department of Nephrology and Lupus/Vasculitis Centre, Srinagar, India

**Keywords:** COVID-19-induced iTTP, Hydoxychloroquineinduced TTP, ADAMTS13, Von Willebrand factor, Thrombotic microangiopathy

## To the Editor,

I read with great interest the recently published letter to the editor by Arıkan et al. [1] about hydroxychloroquine (HCQ)-associated thrombotic thrombocytopenic purpura (TTP) together with some queries raised by Sookaromdee and Wiwanitkit and the original authors’ reply [1,2]. There are some pertinent points to note. First and foremost, HCQ-induced TTP is an extremely rare but nevertheless recognized entity with only a few cases reported in the literature, which have not been thoroughly evaluated. Most of the data regarding this complication have been extrapolated against the background of HCQ’s parent compound, quinine. Quinine-induced and presumably HCQ-induced TTP is characterized by generation of antiplatelet antibodies, which cross-react with the endothelium and cause TTP. However, typical drug-induced TTP, including quinine-induced TTP, is not associated with a critical drop in ADAMTS13 levels or with the presence of an ADAMTS13 inhibitor, but the patient presented by Arıkan et al. had both. This is an important differentiating feature of quinine-induced TTP from immune TTP (iTTP) and it calls into question the diagnosis made by Arıkan et al. [1] or even suggests iTTP after HCQ as a novel complication [3]. I would not straight away repudiate the observation made by Arıkan et al. [1] because, first of all, there is a definite temporal profile that links the medicine with iTTP, and, furthermore, some drugs like the thienopyridine group of antiplatelets have been associated with classic iTTP leading to a critical drop in ADAMTS13 levels and the presence of an inhibitor [4]. At least five cases of thrombotic microangiopathy of iTTP/HUS (hemolytic uremic syndrome) in coronavirus disease-19 (COVID-19) have been reported in the literature with an obvious temporal relation between HCQ and the development of iTTP, which seems to have been ignored by most authors in light of COVID-19 infection and everything being attributed to this novel virus [5,6,7,8]. It needs to be emphasized that quinine-induced thrombotic microangiopathy can occur hours, days, or months after exposure to the drug and HCQ has a very long elimination half-life of 40 days after a single dose. The typical COVID-19-induced TTP-like illness is secondary to diffuse endothelial damage secondary to virus-induced endotheliitis further aggravated by complement overactivity, causing an over-spilling of von Willebrand factor (vWF) from the damaged endothelial network overwhelming the ADAMTS13 protease and producing the relative ADAMTS deficiency typically seen as an ADAMTS13/vWF ratio of <10, but without a critical drop in ADAMTS13 levels and in the absence of a circulating ADAMTS inhibitor [9]. Nevertheless, some cases of classic iTTP have occurred in association with COVID-19 infection, possibly due to some novel mechanism triggered by the virus or maybe the unmasking of a latent disease with the virus possibly acting as a second hit. Differentiating primary from secondary thrombotic microangiopathy is not always easy since many genetic or even acquired variants could be clinically silent, becoming manifest later in life following a second hit such as an infection, drug, surgical stress, or pregnancy. In the case presented by Arıkan et al. [1], I feel that at least immunoglobulin G anti-COVID antibodies should have been examined and, if possible, novel in vitro HCQ sensitization testing should be performed as well [10]. Although re-exposure to the drug could easily solve the diagnostic dilemma, it would be absolutely unethical and risky since the second exposure could be more devastating if the drug were in fact the culprit. The initial illness in the patient could have been any trivial infection or maybe a mild COVID case (though not documented). I am sure the authors must have thoroughly evaluated the patient for other mimickers and ruled them out, and systemic lupus erythematosus seems very unlikely. The role of HCQ in causing iTTP in the given case cannot be absolutely ruled out.
