# Supplementary material for: Immune Thrombotic Thrombocytopenic Purpura in a Patient with Suspected COVID-19: Hydroxychloroquine Culprit or Just Happenstance?
Source: Turk J Haematol. 2021 Jun 1;38(2):155–7. doi: 10.4274/tjh.galenos.2021.2021.0057 (PMC8171205; doi:10.4274/tjh.galenos.2021.2021.0057)
Supplement: Related texts

# REPLY FROM THE AUTHORS

## To the Editor,

We thank our colleague for his interest and excellent comments.

We totally agree that the case that we reported is not a typical case of quinine-induced thrombotic thrombocytopenic purpura (TTP) [1]. As Mir emphasized, a significant decrease in the activity of ADAMTS13 or the presence of inhibitors is not an expected finding in quinine-associated TTP. However, infections as well as drugs can act as a second hit in some patients with silent genetic or acquired TTP variants, as Mir stated. Furthermore, HCQ could act as a second hit in the patient, or HCQ could also cause TTP with low ADAMTS13 activity in some patients, similar to other drugs such as the thienopyridine group of antiplatelet drugs. That is why we assume that this was a case of antibody-mediated TTP, possibly induced by HCQ.

There are some reported cases of severe acute respiratory syndrome causing coronavirus-2 presenting with TTP in the literature. In most of these cases, lung CT showed significant infiltration with positive polymerase chain reaction tests by nasopharyngeal swab [2] or positivity for specific immunoglobulin (Ig) G [3]. Our limitation was that specific IgG could not be determined serologically. In addition, there were no laboratory abnormalities that suggested coronavirus disease-19 (COVID-19), such as lymphopenia or high serum ferritin or fibrinogen. We also excluded cardiovascular, autoimmune, and malignant diseases. After the patient was discharged, we tried to contact the patient, but we learned that this individual had moved to another city. Interestingly, we were informed that the patient was diagnosed with COVID-19 by rapid testing and specific clinical findings in a different hospital 4 months later. At that time, the patient was not using HCQ. The patient improved without significant sequelae. Furthermore, we could not determine specific IgG levels related to the illness complicated with TTP during the first hospitalization.

HCQ has some well-known hematological side effects including anemia, thrombocytopenia, agranulocytosis, leukopenia, and aplastic anemia. There are two case reports from before the COVID-19 pandemic in the literature in terms of possible HCQ-related TTP, but these relations were suspicious because these patients had respective medical histories of systemic lupus erythematosus and rheumatoid arthritis [4,5].

In conclusion, it is not possible to confidently insist that HCQ has a definite role in the etiology of thrombotic microangiopathy, but our observation should be kept in mind as HCQ might be associated with TTP.

**Fatma Arıkan, Yasin Yıldız, Tarık Ercan, Özen Oruç, Seçkin Akçay, Fergun Yılmaz, Tayfur Toptaş, Tülin Tuğlular**

## References

[1] Arıkan F, Yıldız Y, Ercan T, Oruç Ö, Akçay S, Yılmaz F, Toptaş T, Tuğlular T (2020). Hydroxychloroquine-associated thrombotic thrombocytopenic purpura. Turk J Hematol.

[2] Hindilerden F, Yonal-Hindilerden I, Akar E, Kart-Yasar K (2020). COVID-19 associated autoimmune thrombotic thrombocytopenic purpura: report of a case. Thromb Res.

[3] Capecchi M, Mocellin C, Abbruzzese C, Mancini I, Prati D, Peyvandi F (2020). Dramatic presentation of acquired thombotic thrombocytopenic purpura associated with COVID-19. Haematologica.

[4] Fromm LM (2018). Suspected hydroxychloroquine-induced thrombotic thrombocytopaenic purpura. J Pharm Pract Res.

[5] Mar N, Mendoza Ladd A (2011). Acquired thrombotic thrombocytopenic purpura: puzzles, curiosities and conundrums. J Thromb Thrombolysis.

[6] Naranjo CA, Busto U, Sellers EM, Sandor P, Ruiz I, Roberts EA, Janecek E, Domecq C, Greenblatt DJ (1981). A method for estimating the probability of adverse drug reactions. Clin Pharmacol Ther.
